# DigiFuehr 2.0: Novel insights for digital leadership

**DOI:** 10.1002/1348-9585.12383

**Published:** 2023-01-18

**Authors:** Kevin Claassen, Dominique Rodil Dos Anjos, Jan Patrick Kettschau, Sammy Joelle Shirley Wrede, Horst Christoph Broding

**Affiliations:** ^1^ Chair of Occupational Medicine and Corporate Health Management, Faculty of Health, Department of Human Medicine Witten/Herdecke University Witten Germany

**Keywords:** digital leadership, digitization, index, municipal administration, score

## Abstract

**Objectives:**

Against the background of e‐government, it is discussed whether self‐organization represents an independent component of digital leadership. Addressing this research question, the instrument “DigiFuehr” is being expanded to include the aspects of self‐determination and coordination. The aim is to test the model quality of three adapted scales against the already established support‐focusing version.

**Methods:**

Based on an online survey of *n* = 422 employees at visual display unit workstations in German municipal administrations in April 2022, the original version of DigiFuehr as well as one reduced and two extended versions were validated considering their local and global model fit.

**Results:**

All four scales show a good local fit with regard to internal consistency (Cronbach's α), discriminatory power, and dimensionality. Only the scale extended by two items also offers a sufficient global fit and is thus most suitable as a predictor for psychological stress, work satisfaction, and satisfaction with the professional exchange during homeworking.

**Conclusions:**

With “DigiFuehr 2.0”, an improved version of the original scale is available. Self‐organization is not a component independent of the support dimension, but a further important indicator of a latent digital leadership culture. Neither should come at the expense of the other.

## 1 BACKGROUND

In the framework of Work 4.0, there is a firm association between digital leadership, employee productivity, and corporate success. Digital leadership is an approach that describes the appropriate tasks and tools of leadership in times of digital transformation.^1^

It is based on the further development of participative leadership models. In this way, agility and flexibility are promoted more strongly in order to remain capable of action and reaction in a potentially disruptive market or political environment.^2^

Because a single manager alone does not have all the resources needed for this, a change in corporate culture becomes necessary. The result is a flattening of hierarchies, decentralization, and networking in the sense of pluralistic leadership approaches.^3^, ^4^

With person‐ rather than task‐oriented leadership, the formulation of inspiring shared visions, and the importance of flow, there are also similarities to the Positive Leadership approach.^2^

The “DigiFuehr” scale is known as an indicator of digital leadership competence at the visual display unit (VDU) workstation in municipal administrations. It consists of seven items, of which one refers to co‐determination, while the rest is support‐oriented. Each item can be rated on a four‐point Likert scale of which the value four indicates full agreement. The original version of the summative tool “has the character of a suggestion that is intended to initiate a scientific discourse, whereby there is a fundamental openness to modifications and improvements”.^5^ In the meantime, further tools measuring digital leadership were published.^1^, ^6^, ^7^, ^8^ Now, there is also feedback both from professional as well as scientific peers.

Contrary to the initial misunderstanding of some professional users, digital leadership should not be confused with other constructs. It needs to be explained that digital leadership is a comparatively young field of research with definitional ambiguities. This is confirmed by Klein who states that “since most of the companies are still in the beginning of their digital transformation, there is a lack of a common understanding and a standard model of digital leadership”.^7^

Regularly, digital leadership is equated with virtual and e‐leadership. However, the latter merely describes the classic social influence exerted by a superior, but supported by technically advanced communication and information systems that bridge physical distances. Within the framework of this paper, digital leadership is defined as a set of principles that ultimately aim to establish a customer‐centric, technologically advanced business model. In the course of this transformation, the demands on managers, but also on entire organizations and their employees without management responsibility, are changing. Some authors even assume that every single employee will be considered a digital leader in the near future, although mutual digital support remains crucial.^9^

Consequently, the DigiFuehr tool targets digital leadership culture at the team level, which also includes horizontal leadership, rather than evaluating the competence of a single leading or non‐leading individual. At the annual meeting of the German Society of Occupational and Environmental Medicine (DGAUM) it was also elaborated that the representation of self‐organization as a part of digital leadership needs to be extended. Kelso highlights a structure of interaction and mutual feedback loops when he points out that “self‐organization refers to the spontaneous formation of pattern and pattern change in complex systems whose elements adapt to the very patterns of behavior they create”.^10^

The reaction to the DigiFuehr publication fits a passage within the results section of the original paper, which states the following^5^:

While the summative instrument in its current form is indeed selective, homogenous and (rather) one‐dimensional, the item targeting co‐determination deviates from the other components due to a noticeable overlap with an independent second factor that could be extracted from the data using PCA. This is expectable when considering that co‐determination depends on autonomy and participation […]. As a requirement of autonomy, it is crucial how far a digital leader is willing to enable cooperation and self‐organization within his team. On this account, further research is well advised to investigate whether co‐determination should be modelled as an independent component of digital leadership.

For these reasons, an extended version of the published DigiFuehr scale is now available, which is called “DigiFuehr 2.0.” The novel scale takes into account the original dimension of support and adds further items targeting self‐organization. Autonomous self‐organization is itself based on participation (as a preliminary stage of vertical autonomy), self‐determination (or decentralization), and coordination (the need for which results from increased redundancy). Hence, self‐organization in the business context can be described as the ability and authorization of subordinate organizational members to make decisions that affect themselves, while their work tasks overlap.^11^

Each of the three elements of self‐organization—participation, self‐determination, and coordination—is reflected by one item of the DigiFuehr 2.0 index. The item that already targets co‐determination (DigiFuehr 1) is equated with participation. A comparison of the two versions with the formulation of its items as well as the supposed factor structure of the novel version is displayed in Table 1.

**TABLE 1:** DigiFuehr 1.0 and 2.0 item comparison and DigiFuehr 2.0 factor structure

DigiFuehr 1.0	DigiFuehr 2.0	Factor (DigiFuehr 2.0)
“I am involved in decisions that affect my work and my digital work environment.” (DigiFuehr 1)	“I am involved in decisions that affect my work and my digital work environment.” (DigiFuehr 1)	Self‐organization (Participation)
	“I can determine which working methods, procedures and solution approaches I use to process my tasks.” (DigiFuehr 8)	Self‐organization (Self‐determination)
	“I coordinate my work tasks with my colleagues.” (DigiFuehr 9)	Self‐organization (Coordination)
My digital literacy is encouraged by my manager.” (DigiFuehr 2)	My digital literacy is encouraged by my manager.” (DigiFuehr 2)	Support
“When there is a need for questions about digitalization, I receive support from my manager.” (DigiFuehr 3)	“When there is a need for questions about digitalization, I receive support from my manager.” (DigiFuehr 3)	Support
“I get regular feedback on the quality of my digital work.” (DigiFuehr 4)	“I get regular feedback on the quality of my digital work.” (DigiFuehr 4)	Support
“I get all the information I need to do my digital job.” (DigiFuehr 5)	“I get all the information I need to do my digital job.” (DigiFuehr 5)	Support
“I am supported by my manager to better understand and use digital applications.” (DigiFuehr 6)	“I am supported by my manager to better understand and use digital applications.” (DigiFuehr 6)	Support
“In my department, digital working methods are encouraged.” (DigiFuehr 7)	“In my department, digital working methods are encouraged.” (DigiFuehr 7)	Support

The paper at hand now aims to test the original version of the DigiFuehr scale against two extended and one reduced version. In this way, the additional information obtained by integrating the items that represent participation, self‐determination, and coordination is questioned critically. It is also examined whether a multidimensional factor structure underlies the construct of digital leadership in municipal administrations when more information on self‐organizing teams is available.

## 2 METHODS

As part of the second survey wave of the project “Health and Digital Change” (GudW), the *n* = 1319 employees at VDU workstations in municipal administrations of the first wave were invited to participate in another online survey via an e‐mail‐link. The survey was hosted on external servers of the Witten/Herdecke University and was open for 4 weeks in April 2022 for the employees of three model regions in North Rhine‐Westphalia (Germany). After 2 weeks a reminder was sent out. The model regions represent the fifth, third, and second quintile of the German Index of Multiple Deprivation (GIMD), which results in a regional socioeconomic range.^12^

Of the 1319 invited employees, 563 opened the e‐mail‐link and *n* = 521 participated in the online survey (Response Rate, RR = 39.50%). After ex‐post exclusion of employees with own management responsibilities, *n* = 422 “leaded” employees at the VDU workstation of municipal administrations remained. Their sociodemographic is illustrated in Table 2.

**TABLE 2:** Sociodemographic of the participating employees (*n* = 422)

Gender: female	63.52%
Age in years (± standard deviation)	43.89 (± 12.08)
Education: at least university entrance qualification	62.21%
Model region I	39.57%
Model region II	24.41%
Model region III	36.02%
Non‐optimal mental strain	78.66%

Item nonresponse on leadership variables ranged from 15.40 percent (DigiFuehr 8) to 31.52 percent (DigiFuehr 6). As it was lower than on sociodemographic variables (27.25%–33.41%), missing at random (MAR) was assumed. An exception was the information on the model region, which showed no missings because it was set as mandatory.

The missing values were imputed via Multiple Imputation by Chained Equations (MICE) as proposed by Rubin.^13^ The imputation method was Predictive Mean Matching (PMM) of both the sociodemographic as well as leadership variables. Ten datasets were extracted and averaged.

The original DigiFuehr tool had been constructed as a summative scale of the items DigiFuehr 1 to 7 and was defined here as model 1. Model 2 summed up the items DigiFuehr 2 to 7 which means that it was deprived of the item targeting co‐determination (DigiFuehr 1) and thus exclusively represented the factor digital support. While model 3 took the original version and added one item (DigiFuehr 8), model 4 was the full DigiFuehr 2.0 model, which consisted of the items DigiFuehr 1 to 9. The factor self‐organization was represented by one item in model 1 (DigiFuehr 1: participation), two items in model 3 (+ DigiFuehr 8: self‐determination) and three items in model 4 (+ DigiFuehr 9: cooperation). In any case, it was assumed that the items were reflective measures of their underlying constructs. For the sake of illustration, the scale values were again projected onto the value range between 0 and 100.

Thereon, the four DigiFuehr models, and their items, respectively, were tested with regard to local and global model fit. While, the cutoff points for global fit were taken from Hu and Bentler,^14^ diagonally weighted least squares (WLSMV)^15^ were used as an estimator of confirmatory factor analysis due to the ordinal level of the Likert scales. However, the cutoff points for global fit should rather be regarded as “rules of thumb” that do not work sufficiently well in every context.^16^

The local fit testing referred to internal consistency^17^ and selectivity^18^ covering convergent validity. Cronbach's α was expected to be ≥.7. The selectivity coefficient for each item as assessed by the item to total Spearman ρ correlation (to its associated scale after part‐whole correction) was expected to be ≤.3. This first step aimed at the selection of items. It also indicated that a fifth model including the items DigiFuehr 1 to 7 and 9 showed less discriminatory power than model 4.

The local fit testing of discriminant validity was performed by assessing the dimensionality via the Kaiser–Gutmann criterion^19^ stating that every factor with an eigenvalue >1 should be extracted. This second step aimed at examining the dimensionality of the digital leadership construct and the autonomy of the self‐organization factor.

In the third step, the global fit to the data was tested. The exact fit was examined by the significance (*p* ≥ .05) of the *χ* ^2^ goodness of fit.^20^ Additionally, the root mean square error of approximation (RMSEA)^21^ was expected to be ≤0.06 (as *n* > 250) and reported with the 90 percent confidence interval. The cutoff point for the standardized root mean square residual (SRMR) was set to ≤0.08. The two indices were tested in order to quantify the absolute global fit. The Tucker–Lewis index (TLI)^22^ and the comparative fit index (CFI)^14^ tested the relative global fit by comparing the proposed model to a null model with uncorrelated manifest variables. Both indices of relative global fit should be >0.95.

Finally, a concurrent criterion validation was carried out by calculating the Spearman correlation between each of the DigiFuehr models and the Wuppertal screening index of mental strain (WSIB),^23^ overall work satisfaction (OWS) and satisfaction with the “professional exchange with colleagues during homeworking” (PES), respectively. WSIB was coded dichotomously as 0 = “optimal mental strain” and 1 = “non‐optimal mental strain.” Satisfaction was increasing from one to four. A moderate negative correlation (< −.1) between digital leadership and mental strain was assumed as well as a moderate positive correlation (>.1) between digital leadership and OWS as well as PES, respectively. Due to supposedly imperfect reliability of the DigiFuehr constructs, a single reduction correction was performed for every correlation coefficient.^24^ The classification of the level of correlation as effect size was done according to Ellis.^25^

## 3 RESULTS

Table 3 presents the mean and the standard deviation of the nine potential DigiFuehr 2.0 items as well as the scale values of the four models. It can be seen that the extended versions (models 3 and 4) and their items DigiFuehr 8 and 9 yield higher mean values indicating tendencies of higher self‐organization as compared to support in the surveyed teams after circa 1.5 years of Covid‐19 pandemic. Simultaneously, there is less variability of the self‐organization dimension. The results of the model comparisons are presented below in detail and summarized in Table 4. Green color represents a positive test result and red color a negative.

**TABLE 3:** Mean ± standard deviation of the “DigiFuehr” items and scales (*n* = 422)

DigiFuehr 1	2.42 (± 0.78)
DigiFuehr 2	2.50 (± 0.78)
DigiFuehr 3	2.66 (± 0.83)
DigiFuehr 4	2.17 (± 0.79)
DigiFuehr 5	2.61 (± 0.73)
DigiFuehr 6	2.47 (± 0.82)
DigiFuehr 7	2.80 (± 0.79)
DigiFuehr 8	3.21 (± 0.70)
DigiFuehr 9	3.01 (± 0.62)
Model 1^a^	50.63 (± 21.03)
Model 2^b^	51.17 (± 21.78)
Model 3^c^	53.49 (± 19.50)
Model 4^d^	54.99 (± 18.08)

^a^ Items DigiFuehr 1–7.

^b^ Items DigiFuehr 2–7.

^c^ Items DigiFuehr 1–8.

^d^ Items DigiFuehr 1–9.

**TABLE 4:** Validation of the four “DigiFuehr” models

	Model 1^a^	Model 2^b^	Model 3^c^	Model 4^d^	Criterion
Cronbach‘s α	.90	.91	.89	.88	≥.7
Selectivity (minimum)	0.72 (DigiFuehr 1)	0.76 (DigiFuehr 5)	0.41 (DigiFuehr 8)	0.36 (DigiFuehr 9)	≥0.3
Eigenvalues	4.46	4.08	4.58	4.67	>1
Goodness of fit (*χ* ^2^, *df*, *p*)	63.17, 14, .00	40.34, 9, .00	79.08, 20, .00	71.97, 27, .00	*p* ≥ .05
RMSEA	0.09	0.09	0.08	0.06	≤0.06
SRMR	0.04	0.03	0.04	0.04	≤0.08
TLI	0.90	0.92	0.90	0.93	≥0.95
CFI	0.94	0.95	0.93	0.95	≥0.95
Correlation to WSIB	−.24	−.25	−.29	−.30	lρl > .1
Correlation to OWS	.41	.39	.46	.47	lρl > .1
Correlation to PES	.31	.31	.31	.32	lρl > .1

^a^ Items DigiFuehr 1–7.

^b^ Items DigiFuehr 2–7.

^c^ Items DigiFuehr 1–8.

^d^ Items DigiFuehr 1–9.

Cronbach's α ranges from .88 (model 4) to .91 (model 2) indicating a high internal consistency and thus high reliability of every model. The minima of the selectivity coefficients of the items associated with the four models are throughout acceptable (.36–.76). For each model containing six, seven, eight, or nine items, only one principal component with an eigenvalue >1 can be extracted. The single extracted principal components show high eigenvalues between 4.08 for the smallest model (model 2) and 4.67 for the largest (model 4). The corresponding explanation of total item variance is between 68.12 and 51.99 percent. This indicates a one‐dimensional structure of all of the DigiFuehr 2.0 constructs.

At *p* < .001 all four null hypothesis that the modeled equal the empirical covariance matrices have to be rejected. Although, the SRMR of all four models pass the threshold of SRMR ≤0.08 (SRMR = 0.03–0.04). Only model 4 shows a RMSEA ≤ 0.06 (RMSEA = 0.06, 90% CI: 0.05–0.08). While its TLI is just lower than the postulated cutoff point of 0.95 (TLI = 0.93), the CFI of 0.95 indicates an acceptable relative global fit of model 4. Model 2 also shows an acceptable CFI of 0.95.

As expected there is a moderate negative relationship between digital leadership and mental strain (WSIB) (0.24–0.30) and a moderate positive relationship between digital leadership and overall work satisfaction (OWS) (0.39–0.47) as well as satisfaction with professional exchange with colleagues during homeworking (PES) (0.31–0.32). The Spearman correlation is constantly highest for model 4, making it the best predictor with the highest model fit and thus a suitable candidate for DigiFuehr 2.0, although its local fit is slightly lower.

**FIGURE 1:**
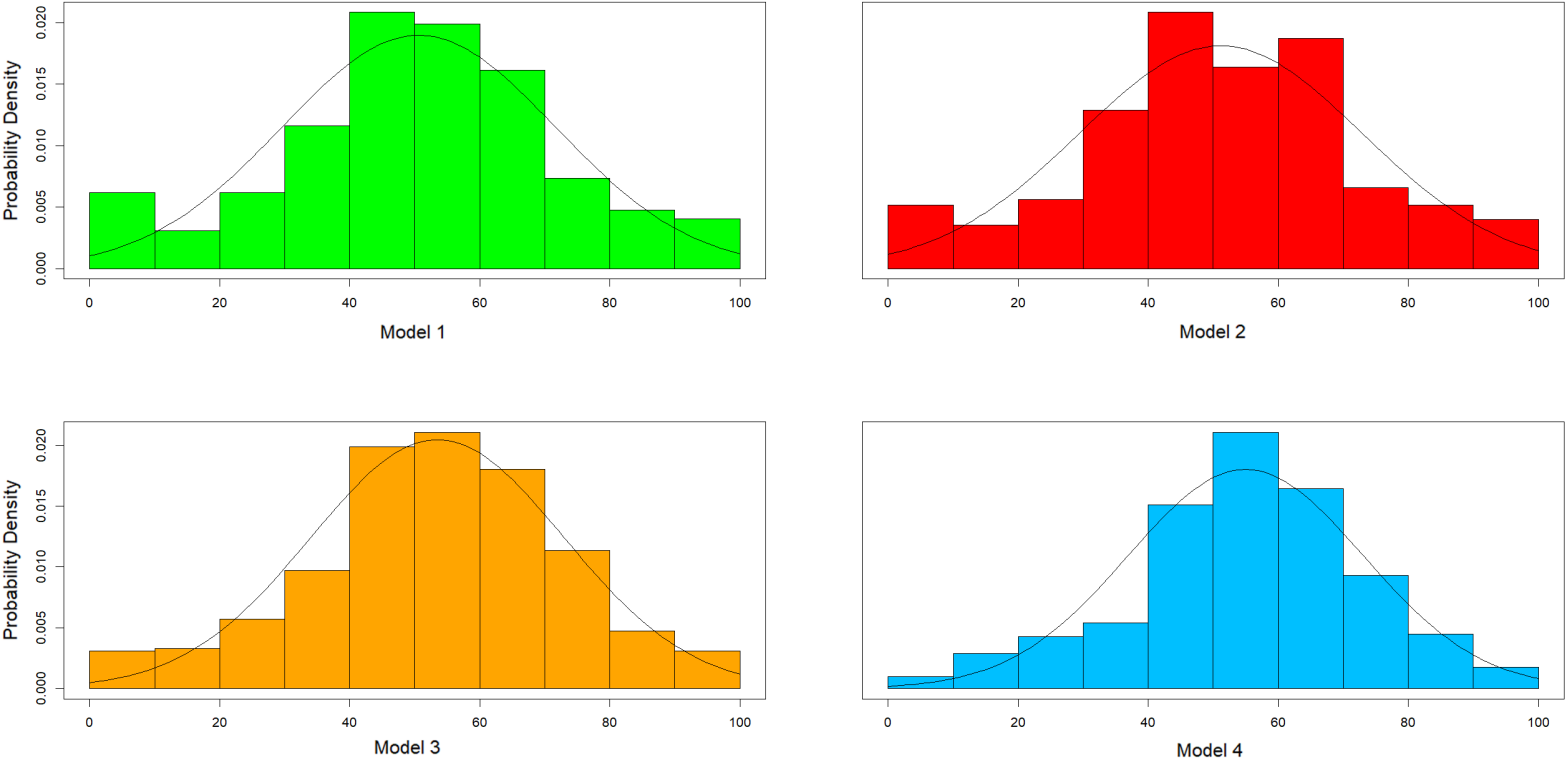
Histograms of DigiFuehr models with normal distribution curve.

All DigiFuehr scales are approximately distributed normally, which can be seen from Figure 1 and is confirmed computationally by Shapiro–Wilk testing (*p* < .001). Different from the results of the first wave, DigiFuehr model 1 shows a fat tail at the left‐hand side, which is eliminated by adding the dimension of self‐organization (models 3 and 4).

Compared to the results of the first wave, the internal consistency of the original DigiFuehr version has slightly increased from α = .88 to α = .90. On the item level, every variable shows higher values and less variance, resulting in higher selectivity of the least performing item DigiFuehr 1 of the original scale (0.47 vs. 0.72) as well as an increased mean scale value at the decreased variance of model 1 (47.33 ± 22.26 vs. 50.63 ± 21.03).

## 4 DISCUSSION

All the tested DigiFuehr models show a good local fit as assessed by internal consistency (Cronbach's α), selectivity (part‐whole corrected item to total correlation), and dimensionality (Kaiser–Gutmann criterion). For model 1, this could already be seen from the original publication. The items of the DigiFuehr scales are based on a one‐dimensional construct, even if the factor self‐organization, which consists of participation, self‐determination, and coordination, is integrated. From that, it can be concluded, that self‐organization—just like the support dimension—is a reflection of digital leadership culture in work teams of municipal administrations rather than an independent part of digital leadership.

However, only model 4 is characterized by an acceptable global fit to the data in terms of RMSEA, SRMR, and CFI. Moreover, it shows the highest correlation to mental strain (WSIB), overall work satisfaction, and satisfaction with professional exchange during homeworking. Following Ellis,^25^ the correlations classify as medium, making digital leadership an acceptable predictor that could be explored in further studies. Furthermore and although self‐organization is not an independent factor, it is obviously an important one that helps to improve the original DigiFuehr scale which already showed a good local fit. Hence, model 4 qualifies as the novel DigiFuehr 2.0 scale. Its usage can be recommended and preferred over the original version.

That none of the models fits the empirical data exactly can be explained by considering the peculiarities of using the *χ* ^2^ statistics as the basis of goodness of fit testing. Babyak and Green^26^ argue with the number of cases, which is *n* = 422 in the present study:

If the sample size is large, the T value will necessarily be large, and even small and possibly unimportant discrepancies between the model implied and observed covariance matrix will yield significance. It is our observation that tests of models are routinely significant – meaning that we conclude our model does not fit – when sample size exceeds 200.

Comparing the validation of the original DigiFuehr scale between the first (October 2020) and second survey wave (April 2022) two facts are striking:

1. The item participation (DigiFuehr 1) shows an improved selectivity.
2. The overall scale value has increased.

This could be traced back to the fact that the digitization has been accelerated by the COVID‐19 pandemic. As the second survey wave started after circa 1.5 years of “social distancing,” learning effects in terms of digital leadership culture have occurred. With regard to the surveyed employees of the three municipal administrations, this is especially true for the self‐organization factor. Its items have much higher scores than the digital support items, which indicates potential for improvement. This particularly refers to the team members at the left tail of the DigiFuehr 1 distribution. Although the digital support factor has increased compared to the first survey wave, digital leaders are well advised to not neglect it for a promotion of self‐organization. This requires tactfulness with regard to situations as well as to the individuality of employees.

Future studies might look at the field of tension between standardization/routinization/control on the one hand and self‐organization/autonomy/employee empowerment on the other. While the digitization of administrative tasks supposedly leads to higher degrees of standardization,^27^ taking a deeper look at the interplay of the two domains might nevertheless be able to resolve apparent contradictions. Gierlich‐Joas et al.^6^ provide a potential framework. A comparison across other working sectors than municipal administrations seems also promising.

As a limitation, it is to mention that support is already a well‐known, well‐studied leadership aspect, and the self‐organization factor also shows overlap with existing concepts such as autonomy^28^ and participative leadership.^29^ Potential added value over these established concepts needs to be tested on additional samples with different outcomes. Until then, the focus is on the finding that digital leadership is a bidimensional construct that goes beyond the dimension of support.

As the DigiFuehr questions were asked in German an intercultural validation is still pending at this point in time, what might also encourage further research. Additionally, we had to handle a substantial amount of unit nonresponse/loss to follow up leading to a potential selection bias. This could be attributed to a shorter survey period. According to the project managers in the municipal administrations however, it was primarily the reception of Ukrainian refugees that led to significant extra work and thus reduced the willingness of employees to participate in the survey. There is no evidence of an association between nonresponse and digital leadership.

The DigiFuehr scale in both versions 1.0 and 2.0 is constructed as a summative scale. Therefore, its application is easy to handle and of practical benefit for occupational health professionals in administrative institutions. Apart from the GudW Project, inter alia a large German university hospital utilizes the DigiFuehr scale in order to (re‐)evaluate the digital leadership culture in its administrative departments. The results can also be part of the corporate risk assessment of psychological stress targeting the factors leadership and new forms of work. This assessment relates to the employer's duty of care and is based on the directive 89/391/EEC of the European Union,^30^ which had to be implemented into the national law of the member states. Company physicians are responsible for advising the employer. They work together with occupational health managers and can use the results of the risk assessment as a starting point for preventive measures (digital health day, etc.).

## 5 CONCLUSIONS

The extension of the original DigiFuehr scale, which already shows a good local fit, to include items on the domains of self‐determination and coordination substantially increases the global fit of the instrument. Consequently, the usage of the improved version DigiFuehr 2.0 is recommended.

Along with participation, self‐determination and coordination are components of self‐organization. However, also within the framework of DigiFuehr 2.0, self‐organization is not a factor independent of support‐oriented leadership, but another important reflection of an underlying digital leadership culture. Neither should come at the expense of the other.

## AUTHOR CONTRIBUTIONS

KC was responsible for the conception as well as the methodology and wrote the original article. DRdA conducted the survey and dealt with data curation. JPK took care of the software, the formal analysis and the visualization. SJSW did text review and validation. HCB's responsibilities included supervision and project administration. All authors read and approved the final manuscript.

## DISCLOSURE

The authors declare that there is no conflict of interest.

## INFORMED CONSENT

Informed consent from all participants was obtained online.

## Data Availability

The data that support the findings of this study are available from the corresponding author upon reasonable request.
